# Longitudinal Study of Cognitive Functioning in Adults with Juvenile Idiopathic Arthritis

**DOI:** 10.3390/biomedicines10071729

**Published:** 2022-07-18

**Authors:** Natalia Mena-Vázquez, Fernando Ortiz-Márquez, Pablo Cabezudo-García, Claudia Padilla-Leiva, Gisela Diaz-Cordovés Rego, Luis Muñoz-Becerra, Teresa Ramírez-García, Jose Manuel Lisbona-Montañez, Sara Manrique-Arija, Arkaitz Mucientes, Esmeralda Núñez-Cuadros, Rocío Galindo Zavala, Pedro Jesús Serrano-Castro, Antonio Fernández-Nebro

**Affiliations:** 1 Instituto de Investigación Biomedica de Málaga (IBIMA)-Plataforma Bionand, 29010 Malaga, Spain; natalia.mena.sspa@juntadeandalucia.es (N.M.-V.); fomquez@gmail.com (F.O.-M.); gisela.d.cordoves@hotmail.com (G.D.-C.R.); psicologo.uecmalaga@gmail.com (L.M.-B.); terramgar@gmail.com (T.R.-G.); josemanuellisbona@hotmail.com (J.M.L.-M.); sarama_82@hotmail.com (S.M.-A.); arkaitzmucientes@gmail.com (A.M.); esmenunez@gmail.com (E.N.-C.); rociogalin@hotmail.com (R.G.Z.); pedro.serrano.c@gmail.com (P.J.S.-C.); afernandezn@uma.es (A.F.-N.); 2 Unidad de Gestion Clinica de Reumatología, Hospital Regional Universitario de Málaga, 29009 Malaga, Spain; 3 Servicio de Neurología, Unidad de Gestion Clinica de Neurociencias, Hospital Regional Universitario de Malaga (HRUM), 29010 Malaga, Spain; 4 Departamento de Medicina, Universidad de Málaga, 29016 Malaga, Spain; claupale@hotmail.es; 5 Unidad de Gestion Clinica de Pediatría, Hospital Regional Universitario de Málaga, 29009 Malaga, Spain

**Keywords:** juvenile idiopathic arthritis, cognitive functions, inflammation, biological therapy

## Abstract

Objective: To prospectively evaluate possible decline of cognitive functions in adult patients with juvenile idiopathic arthritis (JIA) and identify associated factors. Patients and methods: We performed a 24-month prospective observational study of adults (≥16 years) with JIA. The primary outcome measure was decline in cognitive function defined as a worsening of ≥2 points on the scales of the subsets administered to evaluate the different cognitive areas using the Wechsler Adult Intelligence Scale (WAIS) after 24 months: attention/concentration (digit span); verbal function (vocabulary); visual-spatial organization (block design); working memory (letter-number sequencing); and problem solving (similarities). Other variables included average inflammatory activity using C-reactive protein and composite activity indexes, comorbidity, and treatment. Logistic regression was performed to identify factors associated with cognitive decline. Results: The study population comprised 52 patients with JIA. Of these, 15 (28.8%) had cognitive decline at V24. The most affected functions were working memory (17.3%), attention/concentration (9.6%), verbal function (7.7%), visual-spatial organization (7.7%), and problem solving (3.8%). There were no significant differences in the median direct or scale scores for the cognitive functions evaluated between V0 and V24 for the whole sample. The factors associated with cognitive decline in patients with JIA were average C-reactive protein (OR [95% CI], 1.377 [1.060–1.921]; *p* = 0.039), depression (OR [95% CI], 3.691 [1.294–10.534]; *p* = 0.015), and treatment with biologics (OR [95% CI], 0.188 [0.039–0.998]; *p* = 0.046). Conclusion: Cognitive decline was detected in almost one third of adults with JIA after 24 months of follow-up. Systemic inflammatory activity in JIA patients was related to cognitive decline. Patients treated with biologics had a lower risk of decline in cognitive functions.

## 1. Introduction

Juvenile idiopathic arthritis (JIA) comprises the most frequent group of inflammatory rheumatic disorders in children and can lead to major morbidity and disability in the short and long terms [1]. JIA includes heterogeneous inflammatory diseases that have little more in common than arthritis and age of onset, together with a systemic component with variable severity [2,3]. As is the case in adult arthritis, musculoskeletal manifestations predominate in patients with JIA. However, given its systemic inflammatory nature, JIA can affect other organs and, therefore, lead to manifestations such as psychiatric disorders and cognitive impairment [4,5].

Cognitive decline can be defined as the objective worsening of memory and other cognitive functions compared with a previous state. Cognitive decline is a physiological process that occurs with aging but also is pathologic when the worsening is greater than expected for normal aging with different functional repercussions in daily activities (mild cognitive impairment and dementia). Cognitive decline and cognitive impairment are comorbid conditions that can affect functioning and quality of life in patients with chronic joint diseases [6]. Several studies have evaluated cognitive impairment in rheumatoid arthritis (RA) and the factors that may be associated with this comorbid condition [4,6,7,8,9]. In most studies, cognitive impairment has been defined as a neuropsychological test score below standards values but also when scores were lower than in healthy controls. In a recent meta-analysis of 15 cross-sectional observational studies [9], the authors reported a high prevalence of cognitive impairment in patients with RA, ranging from 38% to 71%. Similarly, in their cross-sectional study of a prospective cohort (464 patients with RA), Katchamart et al. [6] found that inflammatory activity was associated with cognitive impairment after a median follow-up of 5 years. The authors proposed that, consistent with other studies in other inflammatory diseases [10], systemic inflammation can affect cognitive function through increased levels of cytokines, such as TNF-alfa and IL-6, leading to direct damage of neural tissue and thus increasing the risk of atherosclerosis [6,7]. Other factors associated with cognitive impairment include age, psychiatric disorders, and the effect of drugs such as corticosteroids [11]. While data on cognitive function specifically in JIA are scarce [12,13], our group recently observed worse results for visual-spatial function in adults with JIA than in healthy controls and found that a lower score was associated with disease duration, inflammatory activity at inclusion (measured using JADAS27), and level of education [13]. One of the limitations of the study was its cross-sectional design, which did not enable us to determine the presence of true cognitive decline, since patients did not present subjective memory complaints with respect to a previous state. Similarly, since no previous test results were available, we were unable to determine whether other factors such as age, persistence of inflammation over time, treatment, and other comorbid conditions (e.g., depression) could play a role in cognitive decline in patients with JIA. Therefore, we performed a study with the following objectives: (1) to carry out a prospective evaluation of cognitive function in adults with JIA; and (2) to identify factors associated with cognitive decline.

## 2. Materials and Methods

### 2.1. Design

We performed a 24-month prospective, observational study of a cohort of patients with JIA. Recruitment ran from September 2019 to February 2022. The study was approved by the Research Ethics Committee of Hospital Regional Universitario de Málaga (HRUM) (Code 2627-N-21). All the subjects gave their written informed consent to participate.

### 2.2. Patients

We recruited patients aged ≥16 years with JIA classified according to the criteria of ILAR 2001 [3] who were in follow-up at the Department of Rheumatology of HRUM and who participated in the original evaluation of cognitive performance (Code 0925-N-19) [13]. In order to avoid interference in the results of the neuropsychological tests, we excluded patients with inflammatory or rheumatic diseases other than JIA (except secondary Sjögren syndrome), patients with previous neurological disease not associated with JIA, patients treated with drugs that affect the central nervous system (e.g., antiepileptic drugs, antidepressants, benzodiazepines, and barbiturates], and those with low scores in the manual dexterity test.

### 2.3. Protocol

Baseline in 2019 (V0) and at 24 months from the initial evaluation (V24) [13], all of the patients recruited underwent the same neuropsychological test battery according to a pre-established protocol [13], and data were recorded from their clinical history.

Two rheumatologists collected the clinical data and physical examination. One of the rheumatologists performed the physical examination of the patients and the other rheumatologist administrated WAIS subtests. The neuropsychological evaluation was corrected jointly by a neurologist and a neuropsychologist. The neuropsychological evaluation was performed using a series of tests following the appropriate manual. These included the Wechsler Adult Intelligence Scale (WAIS-III, second edition in Spanish, TEA Ediciones) and the Beck Depression Inventory-II (BDI-II). All the tests were administered in the same clinic based on the same timetable as the first evaluation. Before administration of the neuropsychological battery, all patients underwent the Nine-Hole Peg Test to assess their manual dexterity.

#### 2.3.1. Wechsler Adult Intelligence Scale

WAIS-III is the fifth version of the Wechsler Adult Intelligence Scale. It comprises 13 subsets divided between 2 scales: the verbal scale, with the subtests vocabulary, similarities, arithmetic, digit span, information, comprehension, and letternumber sequencing; and the performance scale, including picture completion, digit symbol coding, block design, matrix reasoning, picture arrangement, and symbol search [14]. Some of these subtests make it possible to evaluate the following cognitive domains: attention/concentration using digit span [8,15], verbal functioning using vocabulary [8], visual-spatial organization using block design [8], working memory using letter-number sequencing [16] and digit span [17], and reasoning/problem solving using similarities [8,18]. We selected these 5 domains based on the literature [9,19]. We used the second revised edition of WAIS-III in Spanish (TEA Ediciones). The subtests selected were administered and corrected following the guidelines of the corresponding manual for application and correction. Direct (raw) and scaled scores can be obtained for each WAIS-III subtest. Scaled scores are normalized scores for different age groups.

#### 2.3.2. Beck Depression Inventory II (BDI-II)

The Beck Depression Inventory (BDI) 21-item self-assessment instrument is used for evaluating depressive symptoms. According to the score, depression can be classified as normal (0–13), mild (14–19), moderate (20–28), and severe (29–63) [20]. It has proven useful for identifying depression in various medical specialties, including rheumatology [20]. The version used was the Beck Depression Inventory-II (BDI-II) from 1996 [21].

##### Nine-Hole Peg Test

The Nine-Hole Peg Test is used to evaluate manual dexterity. The reference times were those published in Oxford Grice et. al, 2003 [22]. Patients with low scores were considered to have impaired manual dexterity and were excluded from the study [23].

### 2.4. Working Definitions and Variables

The main variable was “cognitive decline”, defined as worsening at 24 months (V24) of ≥2 scale points in a subtest with respect to the score obtained at V0 [24]. The cognitive areas of the corresponding tests were attention/concentration (digit span), verbal function (vocabulary), visual-spatial organization (block design), working memory (letter-number sequencing; and digit span), and problem solving (similarities). Depression was evaluated using the BDI-II, defining the presence of depression when a score of ≥14 is obtained [20].

#### Inflammation

Evaluation of the joints at V0 and V24 included inflammatory activity using the 27-joint Juvenile Arthritis Disease Activity score (JADAS27) [25], and C-reactive protein level (mg/dL) and ESG (mm/h) in blood [26]. We also calculated the average inflammatory activity throughout the follow-up period using the mean JADAS27 and C-reactive protein values (mg/dL) Furthermore, severity-related variables were taken into account, e.g., erosions on radiography and physical function using the Health Assessment Questionnaire (HAQ) [27].

### 2.5. Other Variables

We recorded demographic, laboratory, and treatment-related data. V24 was considered the cut-off for including patients in the study and V0 the baseline date for evaluation of cognitive function in 2019. The demographic and clinical variables included age (years), sex (male/female), educational level (basic, non-university higher, university), body mass index (BMI, weight [kg]/): height [m^2^]), and smoking (active smoker, ex-smoker, and nonsmoker). Traditional cardiovascular risk factors were also recorded. Arterial hypertension was defined as blood pressure ≥140/90 mmHg or treatment with antihypertensive medication [28]. Diabetes mellitus was diagnosed according to the 2010 recommendations of the American Diabetes Association [29]. Dyslipidemia was recorded (LDL cholesterol >160 mg/dL; triglycerides > 200 mg/dL; HDL cholesterol < 40 mg/dL [men] and <50 mg/dL [women]), as was obesity (≥30 kg/m^2^) [30].

The characteristics of patients with a JIA included date of onset of the disease (calculated from the diagnosis to the cut-off), diagnostic delay (months from onset of symptoms to diagnosis of JIA), uveitis, and the following laboratory variables: rheumatoid factor (RF), positive if >20 IU/mL; anticitrullinated peptide antibody (ACPA), positive if >10 IU/mL; HLA27 (positive/negative); antinuclear antibody (ANA), positive if >1/80 at any point during the disease. We recorded all previous drugs, current medication, and changes in medication during the prospective follow-up, including disease-modifying antirheumatic drugs (DMARDs). These included the following: conventional synthetic DMARDs (csDMARDs), such as methotrexate, leflunomide, and sulfasalazine; and biologic DMARDS (bDMARDs), such as tumor necrosis factor inhibitors (anti-TNF), tocilizumab, abatacept, rituximab, ustekinumab, and Janus kinase inhibitors (JAKi [e.g., tofacitinib and baricitinib]). We also recorded corticosteroids.

### 2.6. Statistical Analysis

First, we performed a descriptive analysis of the main patient-related variables. Qualitative variables were expressed as number and percentage. Quantitative variables were expressed as mean ± standard deviation (SD) for normally distributed data and as median ± interquartile range (IQR) for non-normally distributed data. The normality of the distribution of continuous variables was confirmed using the Kolmogorov–Smirnov test. The main variables of cognitive function were compared after 24 months of follow-up (V0 vs. V24). We studied the characteristics of patients with JIA according to the degree of cognitive decline. Hypotheses for qualitative variables were tested using the Pearson χ^2^ test; the *t* test was used for quantitative variables. Finally, we ran a multivariate logistic regression analysis to identify those factors associated with cognitive decline in JIA. We used the same sample as in the evaluation in 2019, whose size was calculated based on data for patients with RA, owing to the lack of data in adults with JIA [31]. Data were entered and the statistical analysis performed using R Commander (John Fox; Hamilton, ON, Canada).

## 3. Results

### 3.1. Clinical Characteristics

Between September 2019 and February 2022, we prospectively followed 52 patients with JIA. There were no losses during recruitment or follow-up or exclusions (Supplementary Figure S1). During follow-up, none of the patients developed a neurological disorder or started treatment with antiepileptic drugs, antidepressants, benzodiazepines, or barbiturates. None of the patients reported subjective cognitive complains. All the patients completed the Nine-Hole Peg Test to assess manual dexterity in less than 5 min, with a mean (SD) time of 14.0 (2.0) s.

The main characteristics at V0 and at V24 are shown in Table 1. In addition, a detailed description of the baseline characteristics of these patients has already been described [13]. Most participants were women (67%), and at V24, the mean (SD) age was 25.0 (6.1) years. At the end of follow-up (vs. V0), more patients were educated to a university level (*p* = 0.001), and although no differences were observed for most cardiovascular risk factors, more patients were smokers (*p* = 0.010) and were overweight or obese (*p* = 0.015). As for clinical-laboratory characteristics, more than half of the patients had longstanding oligoarticular JIA, and 40% had positive ANA titers. There were no significant differences at V24 compared with V0 in inflammatory activity assessed using JADAS27 (*p* = 0.751). The patients had a median JADAS27 of around 4.0 and an average CRP of 3.4 mg/dL (Table 1).

At V24, 20/52 patients (38.4%) were receiving monotherapy with bDMARDs, 13/52 (25.0%) were receiving monotherapy with csDMARDs, 11/52 patients (21.1%) were receiving combination therapy, and 8/52 patients (15.3%) were not receiving DMARDs because their symptoms were controlled (Table 1). There were no significant differences at V24 compared with V0 in the number of patients who were receiving csDMARDs (*p* = 0.785), bDMARDs (*p* = 0.182), or corticosteroids (*p* = 0.103). During follow-up, two patients suspended leflunomide owing to intolerance, and another patient switched leflunomide for sulfasalazine because she wished to become pregnant, although she did not become pregnant during this period. As for bDMARDs, three patients started a new treatment: one patient initiated adalimumab, one certolizumab, and one secukinumab. One patient discontinued tofacitinib owing to inefficacy and switched to golimumab.

### 3.2. Prospective Evaluation of Cognitive Function

Table 2 shows the numerical differences in cognitive function evaluated at V0 and V24 for the whole sample. There were no significant differences in the median direct or scale scores for the cognitive functions evaluated between V0 and V24. At the end of follow-up, the values recorded for depression based on the BDI questionnaire were higher (*p* = 0.035), and more patients fulfilled the criteria for depression (*p* = 0.046).

A total of 15 patients (28.8%) had cognitive decline with worsening ≥2 scale points in the subtests administered to evaluate different cognitive areas. Cognitive decline was recorded for 10/15 patients (66.6%) in 1 subtest, for 4/15 patients (26.6%) in 2 subtests, and 1/15 patients (6.6%) in 3 subtests. The cognitive tests showing decline with respect to V0 in a larger percentage of patients were digit span (11.5%) and letter-number sequencing (9.6%), followed by vocabulary (7.7%), block design (7.7%), and, finally, similarities (3.8%) (Table 2). Considering the functions evaluated by these tests, the cognitive functions most affected were working memory (17.3%), followed by attention/concentration (9.6%), verbal function (7.7%), visual-spatial organization (7.7%), and finally problem solving (3.8%). There were no significant differences in the median direct or scale scores for the cognitive functions evaluated between V0 and V24 for the whole sample (Supplementary Figure S2). The characteristics of patients with JIA and cognitive decline are shown in Supplementary Table S1. Among patients without cognitive decline, four of them improved in at least one domain (improvement in 2 or more scaled points). Three of these patients were already on anti-TNF in V0 and the other one began anti-TNF before V24. There were no significant variations in CRP between V0 and V24 in these patients. One patient had mild depression in V0 resolved in V24.

### 3.3. Factors Associated with Cognitive Decline in JIA

Table 3 shows the characteristics of patients with JIA at baseline (V0) and the end of follow-up (V24) according to cognitive decline. Fifteen patients (28.8%) with JIA had cognitive decline, with worsening of ≥2 scale points on the subtests administered to evaluate the different cognitive areas. Compared to patients who did not have cognitive decline, those who did were less likely to have a secondary/university education at baseline (60% vs. 84%; *p* = 0.045), had a higher CRP value at baseline (3.0 [2.9–5.0] vs. 2.9 [2.0–2.9]; *p* = 0.048), and were less frequently taking biologics (33.3% vs. 62.2%; *p* = 0.046).

At the end follow-up, (V24) were found to have significant differences in educational level (*p* = 0.030), with more patients educated to secondary and university level among those who did not experience cognitive decline than among those who did. Furthermore, compared to the other patients, those with cognitive decline had a higher median (IQR) BMI (*p* = 0.032), a higher average CRP (*p* = 0.002), and a higher average JADAS27 (*p* = 0.048); fewer of these patients were receiving biologics (*p* = 0.016). Depression was more frequent in patients with cognitive decline (*p* = 0.001) and BDI scores were higher (*p* = 0.027).

In patients with cognitive decline compared with the other patients, the median (IQR) of corticosteroids (grams) did not show significant differences with the baseline values (5.0 [0.0–7.5] vs. 5.0 [0.0–7.5]; *p* = 0.980), or at the end of follow-up (5.0 [0.0–7.5] vs. 5.0 [0.0–5.0]; *p* = 0.548).

### 3.4. Multivariate Analysis

Table 4 shows the results of the multivariate logistic regression analysis in 52 patients with JIA (DV: cognitive decline). The event cognitive decline was recorded in 15/52 patients. During follow-up, cognitive decline was independently associated with more pronounced inflammatory activity (in terms of C-reactive protein) and with depression (as assessed using the BDI). Cognitive decline was less likely in patients receiving biologics. The results were similar in other multivariate regression model excluding seven patients with treatment change during follow-up (Supplementary Table S2).

## 4. Discussion

We performed a prospective evaluation of cognitive function in 52 adults with JIA after 24 months of follow-up. Our objectives were to determine whether cognitive function declines over time and to identify the factors involved. We defined cognitive decline as a worsening of ≥2 scale points in the subtests administered to evaluate the various cognitive areas on the WAIS-III [24]. Consistent with this approach, we found that 28% of patients with JIA had cognitive decline after 24 months of follow-up. At the time of writing, we have found no studies that prospectively evaluated cognitive decline in patients with JIA. In RA, most studies have evaluated the prevalence of cognitive impairment in a cross-sectional manner, with findings ranging from 31% to 71% [9]. Similarly, other studies that have analyzed prospective RA cohorts over 5–20 years of follow-up report cognitive impairment in 24% to 70% of patients [6,32]. These differences are mainly due to the tools used to evaluate cognition. In addition, we must remember that in patients with inflammatory arthritis and a mean age >50 years, other factors, such as cerebrovascular or degenerative disease may play a role. In our study, on the other hand, these factors had little or no effect, since the patients were adults with JIA aged <50 years.

Recent studies have revealed a greater risk of cognitive impairment in rheumatic diseases, with chronic systemic inflammation considered to be the trigger [33,34]. In this sense, the present study shows that average inflammatory activity measured using C-reactive protein throughout follow-up was associated with cognitive decline in patients with JIA. This association has not been evaluated to date in JIA, although the studies on RA by Wallin et al. [32], Katchamart et al. [6], and Lee et al. [35] revealed a significant association between cognitive impairment and inflammatory activity measured using C-reactive protein. The increase in levels of inflammatory cytokines and proteins such as C-reactive protein, TNF alfa, and IL-6 has a negative effect on cognitive function in RA [7]. These markers have also been reported to be elevated in JIA and, as such, could explain the morbidity observed [36]. Based on our evaluation of various inflammatory parameters, we found a stronger association between cognitive decline and C-reactive protein than with clinical activity indexes such as JADAS27. The activity index JADAS27 is the most used in JIA [25,37]. Shin et al. [15] also report that acute phase reactants, such as C-reactive protein, could prove more accurate when establishing the association between cognitive impairment and inflammation, since clinical activity indexes are affected by subjective parameters. As for the domains and functions evaluated in our study, the most effective cognitive domains were working memory and attention, which belong to the executive functions. Impairment in these domains is associated with dysfunction of the prefrontal cortex and alterations in the fronto-parieto-temporal circuit [6]. Katchamart et al. [6] and Lee et al. [35] also reported an association between impairment of executive function and inflammatory activity in RA.

Similarly noteworthy was that fact that patients with JIA receiving biologics were less likely to have cognitive decline. While this association had not been previously assessed in JIA, Chou et al. [38] showed that patients with RA treated with anti-TNF agents (infliximab, etanercept, and adalimumab) had a lower risk of cognitive impairment. However, no association was observed between the use of csDMARDs and cognitive impairment in these patients. In fact, one study showed that the anti-inflammatory effect of anti-TNF agents on cognition could arise before the effects on joint inflammation [39]. Rech et al. [39] studied patients with RA who started treatment with anti-TNF agents. Using functional magnetic resonance imaging, the authors found that cerebral activation diminished during the first 3 days of treatment, whereas joint inflammation was less common from day 28 onward. Therefore, the effect of biologics on cognitive impairment could be a direct central effect and may occur before treatment of joint involvement [40].

We also found that depression was associated with cognitive decline in patients with JIA. Other studies report a possible association between cognitive impairment and depression in RA [41]. Problems affecting concentration and executive functions may be caused by a depressive state, and this could lead to cognitive impairment [9]. Furthermore, in patients with inflammatory arthritis and autoimmune diseases, the frequency of depression has been reported to be increased [4,42,43,44], possibly as a result of various mechanisms, such as high levels of inflammatory mediators that negatively affect monoaminergic neural transmission, maintenance of synaptic plasticity, and physical inability to carry out activities of daily living owing to pain and inflammation [4]. In patients with JIA, fatigue and sleep problems are common [45]. Along similar lines, a recent systematic review [46] revealed a prevalence of anxiety and depression of 7–64% in adults with JIA, finding that this considerably affected quality of life [47]. Of note, 11 patients (21%) in our study had depression. This percentage was significantly higher at the end of follow-up than at V0 (2019). While this finding can be explained by the factors mentioned above, the SARS-CoV-2 pandemic must have played an important role during follow-up. In fact, some studies show that the SARS-CoV-2 pandemic has had a clear impact on the mental health of and psychosocial factors affecting patients with inflammatory arthritis [46,48]. This could also partly explain why there were more smokers, more obese patients, and more patients educated to university level at V0 than at V24. In addition, the most populated age group in our study was that of adolescents.

Our study is subject to a series of limitations. First, we have not included a control group, so we have not been able to evaluate variability scores between JIA patients and the general population and the use of direct (raw) scores for the evaluation of cognitive decline. However, we have been able to describe our main objective of observing cognitive changes in the group of patients with JIA and associated factors. Albeit there were no significant changes in overall cognitive function of the sample, we found an individual cognitive decline in several patients that was significantly associated with several variables. To avoid variance bias in psychometric testing (aging, external factors in the moment of test application, etc.), we used a scaled score (adjusted by age) and considered decline when the variation is of 2 or more points and not in a single scaled score. Also, the clinical and treatment between V0 and V24 clearly can affect the neurological response; for this, we calculated the average inflammatory activity throughout the follow-up period using the mean JADAS27 and C-reactive protein values (mg/dL), and only seven patients (13%) changed treatment during follow-up. As we were unable to compare our results with those of similar studies, we made the comparison using data for other types of chronic arthritis, such as RA. Nevertheless, our study is the first to evaluate prospectively cognitive function in adults with JIA and these data are interesting to consider future clinical trials of biological drugs evaluating cognitive function in these patients. A very long-term follow-up would be recommended to determine if these patients present a higher risk for mild cognitive impairment or dementia. Additionally, it would be interesting if patients with cognitive decline improve in the follow-up and are related to improvement in RPC values or the use of anti-TNF. We must also remember that the SARS-CoV-2 pandemic may have led to greater impairment of patients’ mood, although we identified other variables that were independently associated with cognitive decline in affected patients.

## 5. Conclusions

In conclusion, almost one third of adult patients with JIA had cognitive decline after 24 months of follow-up. Executive functions seem to be more affected in this population. In adults with JIA, cognitive decline was directly associated with average inflammatory activity measured based on C-reactive protein and with depression. Decline was less frequent in patients receiving biologics. Our findings highlight that management of joint involvement should be accompanied by management of inflammatory activity and mental health. Given that inflammation may initiate and maintain cognitive decline in patients with JIA, cognitive functioning should be also assessed in these patients. Future studies should be performed to determine the mechanism of how inflammation affects cognition in young patients with JIA.

## Figures and Tables

**Table 1:** Characteristics of 52 patients with JIA at baseline and at 24 months.

Variable	Baseline *n* = 52	24 Months *n* = 52	*p* Value
Epidemiological characteristics			
Age in years, mean (SD)	22.8 (5.1)	25.0 (6.1)	<0.001
Female sex, *n* (%)	35 (67.3)	35 (67.3)	1.000
Caucasian race, *n* (%)	50 (96.2)	50 (96.2)	1.000
Smoking			0.010
Nonsmoker, *n* (%)	46 (88.5)	38 (73.1)	
Smoker, *n* (%)	6 (11.5)	14 (26.1)	
Educational level			0.001
Basic, *n* (%)	12 (23.1)	5 (9.6)	
Non-university higher, *n* (%)	29 (55.8)	22 (42.3)	
University, *n* (%)	11 (21.2)	25 (48.1)	
Comorbid conditions			
AHT, *n* (%)	1 (1.9)	1 (1.9)	1.000
DM, *n* (%)	1 (1.9)	1 (1.9)	1.000
Dyslipidemia, *n* (%)	2 (3.8)	4 (7.7)	0.259
BMI (kg/m^2^), mean (SD)	22.3 (3.4)	23.8 (4.6)	0.005
Normal (18.5–24.9), *n* (%)	45 (86.5)	39 (75.0)	0.015
Overweight (≥25–<30), *n* (%)	5 (9.6)	6 (11.5)	0.015
Obesity (≥30), *n* (%)	2 (3.8)	7 (13.5)	0.015
Clinical-laboratory characteristics			
Duration of JIA, months, median (IQR)	134.1 (95.6–214.2)	161.4 (103.6–220.4)	<0.001
Diagnostic delay, months, median (IQR)	3.0 (2.3–3.0)	3.0 (2.3–3.0)	1.000
Type of JIA			0.850
Systemic, *n* (%)	3 (5.8)	3 (5.8)	
Oligoarticular, *n* (%)	30 (57.7)	30 (57.7)	
Polyarticular RF+, *n* (%)	1 (1.9)	1 (1.9)	
Polyarticular RF−, *n* (%)	8 (15.4)	8 (15.4)	
Psoriatic, *n* (%)	3 (5.8)	3 (5.8)	
Arthritis-enthesitis, *n* (%)	7 (13.5)	7 (13.5)	
Undifferentiated, *n* (%)	0 (0.0)	0 (0.0)	
Erosions, *n* (%)	8 (15.4)	9 (17.3)	0.322
RF > 10 U/mL, *n* (%)	2 (3.8)	2 (3.8)	1.000
ACPA > 20 U/mL, *n* (%)	2 (3.8)	2 (3.8)	1.000
HLA B27+, *n* (%)	9 (17.3)	9 (17.3)	1.000
ANA, *n* (%)	21 (40.4)	21 (40.4)	1.000
Uveitis, *n* (%)	10 (19.2)	11 (21.1)	0.322
JADAS27, median (IQR)	3.9 (2.9–10.0)	4.0 (2.9–8.3)	0.751
Average JADAS27, median (IQR)	-	4.0 (2.8–9.2)	-
CRP (mg/dL), median (IQR)	2.9 (2.0–2.9)	3.0 (2.9–4.0)	0.228
Average CRP (mg/dL), median (IQR)	-	3.4 (2.9–4.2)	-
HAQ, median (IQR)	0.0 (0.0–0.15)	0.0 (0.0–0.15)	0.285
Average HAQ, median (IQR)	-	0.0 (0.017)	-
Treatment			
Conventional synthetic DMARDs, *n* (%)	24 (46.2)	22 (42.3)	0.785
Methotrexate, *n* (%)	18 (30.8)	18 (30.8)	1.000
Leflunomide, *n* (%)	4 (7.7)	1 (1.9)	0.101
Sulfasalazine, *n* (%)	1 (1.9)	2 (3.8)	0.323
Hydroxychloroquine, *n* (%)	1 (1.9)	1 (1.9)	1.000
Biologic DMARDs, *n* (%)	28 (53.8)	31 (59.6)	0.182
Etanercept, *n* (%)	7 (13.5)	7 (13.5)	1.000
Adalimumab, *n* (%)	12 (23.1)	13 (25.0)	0.780
Golimumab, *n* (%)	1 (1.9)	2 (3.8)	0.323
Certolizumab, *n* (%)	0 (0.0)	1 (1.9)	0.322
Tocilizumab, *n* (%)	7 (13.5)	7 (13.5)	1.000
Tofacitinib, *n* (%)	1 (1.9)	0 (0.0)	0.323
Secukinumab, *n* (%)	0 (0.0)	1 (1.9)	0.323
Corticosteroids, *n* (%)	8 (15.3)	6 (11.5)	0.103
Dose of corticosteroids (grams), median (IQR)	5.0 (0.0–7.5)	5.0 (0.0–6.8)	0.172

Abbreviation: JIA: juvenile idiopathic arthritis; SD: standard deviation; AHT: arterial hypertension, DM: diabetes mellitus; BMI: body mass index; IQR: interquartile range; RF: rheumatoid factor; ACPA: anticitrullinated peptide antibody; HLA: human leukocyte antigen; ANA: antinuclear antibodies; JADAS: Juvenile Arthritis Disease Activity score; CRP: C-reactive protein; HAQ: Health Assessment Questionnaire; DMARD: disease-modifying antirheumatic drugs.

**Table 2:** Cognitive function at baseline and at 24 months in patients with JIA.

Variable	Baseline *n* = 52	24 Months *n* = 52	*p* Value
Verbal functioning			
Vocabulary, direct score, median (IQR)	20.5 (13.0–27.0)	21.0 (17.0–25.7)	0.185
Vocabulary, scale score, median (IQR)	3.0 (3.0–5.0)	4.0 (3.0–5.0)	0.445
Decline ≥2 scale points, *n* (%)	-	4 (7.7)	-
Problem-solving function			
Similarities, direct score, median (IQR)	15.0 (12.0–18.0)	14.0 (12.0–17.0)	0.359
Similarities, scale score, median (IQR)	7.0 (6.0–9.0)	7.0 (6.0–8.0)	0.347
Decline ≥2 scale points, *n* (%)	-	2 (3.8)	-
Attention function			
Digit span, direct score, median (IQR)	14.0 (12.0–16.0)	14.0 (13.0–17.5)	0.191
Digit span, scale score, median (IQR)	8.0 (6.0–10.0)	8.0 (7.0–10.7)	0.119
Decline ≥2 scale points, *n* (%)	-	6 (11.5)	-
Working memory function			
Letter-number sequencing function, direct score, median (IQR)	8.0 (7.0–11.0)	8.0 (6.2–9.7)	0.901
Letter-number sequencing function, scale score, median (IQR)	6.6 (5.0–10.0)	6.5 (6.5–9.7)	0.351
Decline ≥2 scale score, *n* (%)	-	5 (9.6)	-
Visual-spatial organization			
Block design, direct score, median (IQR)	28.0 (24.0–38,)	27.0 (20.2–39.0)	0.102
Block design, scale score, median (IQR)	5.0 (4.0–8.0)	5.0 (4.0–7.0)	0.371
Decline ≥2 scale points, *n* (%)		4 (7.7)	
BDI number, mean (SD)	5.2 (3.9)	6.4 (4.4)	0.035
Depression according to BDI, *n* (%)			0.046
Normal (0–13)	44 (84.6)	39 (75.0)	
Mild (14–19)	4 (7.7)	7 (13.5)	
Moderate (20–28)	3 (5.8)	5 (9.6)	
Severe (≥29)	1 (1.9)	1 (1.9)	

Abbreviations: JIA: juvenile idiopathic arthritis; SD: standard deviation; IQR: interquartile range; BDI: Beck Depression Inventory.

**Table 3:** Characteristics of patients with JIA at baseline and the end of follow-up according to cognitive decline.

Variable	Cognitive Decline *n* = 15		No Cognitive Decline *n* = 37		Cognitive vs. No Cognitive Decline *p* Value	
	Baseline	End of Follow-Up	Baseline	End of Follow-Up	Baseline	End of Follow-Up
Epidemiological characteristics						
Age in years, mean (SD)	21.3 (5.9)	23.5 (6.0)	20.6 (6.3)	23.1 (6.4)	0.718	0.807
Female sex, *n* (%)	10 (66.7)	10 (66.7)	25 (67.6)	25 (67.6)	0.950	0.950
Caucasian race, *n* (%)	14 (93.3)	14 (93.3)	36 (97.3)	36 (97.3)	0.501	0.501
Smoking					0.796	0.979
Nonsmoker, *n* (%)	13 (86.7)	11 (73.3)	33 (89.2)	27 (73.0)		
Smoker, *n* (%)	2 (13.3)	4 (26.7)	4 (10.8)	10 (27.0)		
Educational level					0.045	0.030
Basic, *n* (%)	6 (40.0)	4 (26.7)	6 (16.0)	2 (5.4)		
Non-university higher, *n* (%)	0 (0.0)	11 (73.3)	0 (0.0)	35 (94.6)		
Higher and university, *n* (%)	9 (60.0)	0 (0.0)	31 (84.0)	0 (0.0)		
Comorbid conditions						
AHT, *n* (%)	0 (0,0)	0 (0,0)	1 (2.7)	1 (2.7)	0.520	0.520
DM, *n* (%)	0 (0,0)	0 (0,0)	1 (2.7)	1 (2.7)	0.520	0.520
Dyslipidemia, *n* (%)	0 (0.0)	1 (6.9)	2 (5.4)	3 (8.1)	0.358	0.860
BMI (kg/m^2^), mean (SD)	22.9 (3.7)	25.9 (5.8)	22.0 (3.4)	22.9 (3.8)	0.429	0.032
Normal (18.5–24.9), *n* (%)	12 (80.0)	9 (60.0)	33 (89.2)	30 (81.1)	0.560	0.181
Overweight (≥25–<30), *n* (%)	2 (13.3)	2 (13.3)	3 (8.1)	4 (10.8)	0.560	0.181
Obesity (≥30), *n* (%)	1 (6.7)	4 (26.7)	1 (2.7)	3 (8.1)	0.560	0.181
Clinical-laboratory characteristics						
Duration of JIA, months, median (IQR)	137.3 (73.8–211.7)	168.2 (104.6–239.6)	131.8 (77.0–170.1)	162.6 (100.7–196.9)	0.784	0.857
Diagnostic delay, months, median (IQR)	3.0 (2.9–3.4)	3.0 (2.9–3.4)	3.0 (2.0–3.0)	3.0 (2.0–3.0)	0.620	0.620
Type of JIA					0.233	0.233
Systemic, *n* (%)	0 (0.0)	0 (0.0)	3 (8.1)	3 (8.1)		
Oligoarticular, *n* (%)	11 (73.3)	11 (73.3)	19 (51.4)	19 (51.4)		
Polyarticular RF+, *n* (%)	1 (6.7)	1 (6.7)	0 (0.0)	0 (0.0)		
Polyarticular RF−, *n* (%)	1 (6.7)	1 (6.7)	7 (18.9)	7 (18.9)		
Psoriatic, *n* (%)	0 (0.0)	0 (0.0)	3 (8.1)	3 (8.1)		
Arthritis-enthesitis, *n* (%)	2 (13.3)	2 (13.3)	5 (13.5)	5 (13.5)		
Erosions, *n* (%)	2 (13.3)	2 (13.3)	6 (16.2)	7 (18.9)	0.794	0.630
RF >10 U/mL, *n* (%)	1 (6.7)	1 (6.7)	1 (2.7)	1 (2.7)	0.501	0.501
ACPA >20 U/mL, *n* (%)	1 (6.7)	1 (6.7)	1 (2.7)	1 (2.7)	0.501	0.501
HLA B27+, *n* (%)	3 (20.1)	3 (20.1)	6 (16.2)	6 (16.2)	0.412	0.412
ANA, *n* (%)	7 (46.1)	7 (46.1)	14 (37.8)	14 (37.8)	0.557	0.557
Uveitis, *n* (%)	1 (6.7)	1 (6.7)	8 (21.6)	9 (24.3)	0.197	0.143
JADAS27, median (IQR)	4.9 (2.9–10.0)	4.1 (2.9–9.5)	4.0 (2.9–10.4)	4.0 (2.9–8.9)	0.430	0.426
Average JADAS27, median (IQR)	-	5.6 (2.8–10.0)	-	3.8 (2.6–5.5)	-	0.048
HAQ, median (IQR)	0.0 (0.0–0.2)	0.0 (0.0–0.7)	0.0 (0.0–0.0)	0.0 (0.0–0.09	0.592	0.581
Average HAQ, median (IQR)	-	0.1 (0.0–0.2)		0.0 (0.0–0.1)		0.130
CRP (mg/dL), median (IQR)	3.0 (2.9–5.0)	3.5 (2.9–9.5)	2.9 (2.0–2.9)	2.9 (2.9–4.0)	0.048	0.021
Average CRP (mg/dL), median (IQR)	-	6.5 (3.4–10.2)	-	3.1 (2.9–4.0)	-	0.002
Treatment						
csDMARD, *n* (%)	7 (13.3)	5 (33.3)	16 (43.2)	17 (45.9)	0.670	0.404
bDMARD, *n* (%)	5 (33.3)	5 (33.3)	23 (62.2)	26 (70.3)	0.046	0.016
Corticosteroids, *n* (%)	2 (13.3)	1 (6.7)	8 (21.6)	5 (13.5)	0.492	0.352
Dose of corticosteroids (grams), median (IQR)	5.0 (0.0–7.5)	5.0 (0.0–7.5)	5.0 (0.0–7.5)	5.0 (0.0–5.0)	0.980	0.548
BDI, *n*, mean (SD)	6.4 (4.4)	8.5 (4.9)	4.7 (3.6)	5.5 (4.0)	0.175	0.027
Depression by BDI, *n* (%)					0.179	0.001
Normal (0–13)	11 (73.3)	6 (40.0)	33 (89.2)	33 (89.2)		
Mild (14–19)	3 (20.0)	5 (33.3)	1 (2.7)	3 (8.1)		
Moderate (20–28)	1 (6.7)	3 (20.1)	2 (5.4)	1 (2.7)		
Severe (≥29)	0 (0.0)	1 (6.7)	1 (2.7)	0 (0.0)		

Abbreviations: JIA: juvenile idiopathic arthritis; SD: standard deviation; IQR: interquartile range; AHT: arterial hypertension; DM: diabetes mellitus; BMI: body mass index; ACPA: anticitrullinated peptide antibody; HLA: human leukocyte antigen; ANA: antinuclear antibody; JADAS: Juvenile Arthritis Disease Activity score; CRP: C-reactive protein; HAQ: Health Assessment Questionnaire; csDMARD: conventional synthetic disease-modifying antirheumatic drug; bDMARD: biological DMARD.

**Table 4:** Univariate and multivariate logistic regression of the characteristics of JIA associated with cognitive decline.

Variable	Univariate HR (95% CI)	Multivariate HR (95% CI)	*p* Value
Age, years	0.808 (0.921–1.112)		
Female sex	0.960 (0.268–3.436)		
Educational level *	0.157 (0.025–0.977)		
Body mass index (kg/m^2^)	1.152 (1.004–1.323)		
Average C-reactive protein (mg/dL)	1.392 (1.089–1.779)	1.377 (1.060–1.921)	0.039
Treatment with biologics	0.212 (0.059–0.764)	0.188 (0.039–0.998)	0.046
Depression by BDI ≥ 14	3.338 (1.357–8.213)	3.691 (1.294–10.534)	0.015

* Educational level: higher or university vs. basic. Nagelkerke R^2^ = 0.45. The variables included in the equation were age, sex, educational level, body mass index, C-reactive protein, treatment with biologics, depression by BDI ≥ 14.

## Data Availability

Data presented in this study are available on request from the corresponding author.

## Supplementary Materials

The following supporting information can be downloaded at: https://www.mdpi.com/article/10.3390/biomedicines10071729/s1, Figure S1: Patient flowchart; Figure S2: Scaled Scores; Table S1: Characteristics of patients with JIA and cognitive decline; Table S2: Univariate and multivariate logistic regression of the characteristics of JIA associated with cognitive decline excluding 7 patients with treatment change.

## References

[1] Prakken B., Albani S., Martini A. (2011). Juvenile idiopathic arthritis. Lancet.

[2] Barut K., Adrovic A., Sahin S., Kasapcopur O. (2017). Juvenile Idiopathic Arthritis. Balkan Med. J..

[3] Petty R.E., Southwood T.R., Manners P., Baum J., Glass D.N., Goldenberg J., He X., Maldonado-Cocco J., Orozco-Alcala J., Prieur A.-M. (2004). International League of Associations for Rheumatology classification of juvenile idiopathic arthritis: Second revision, Edmonton, 2001. J. Rheumatol..

[4] Sturgeon J.A., Finan P.H., Zautra A.J. (2016). Affective disturbance in rheumatoid arthritis: Psychological and disease-related pathways. Nat. Rev. Rheumatol..

[5] Veeranki S.P., Downer B., Jupiter D., Wong R. (2017). Arthritis and Risk of Cognitive and Functional Impairment in Older Mexican Adults. J. Aging Health.

[6] Katchamart W., Narongroeknawin P., Phutthinart N., Srinonprasert V., Muangpaisan W., Chaiamnauy S. (2019). Disease activity is associated with cognitive impairment in patients with rheumatoid arthritis. Clin. Rheumatol..

[7] Petersen L.E., Baptista T.S.A., Molina J.K., Motta J.G., do Prado A., Piovesan D.M., de Nardi T., Viola T.W., Vieira E.L.M., Teixeira A.L. (2018). Cognitive Impairment in Rheumatoid Arthritis: Role of Lymphocyte Subsets, Cytokines and Neurotrophic Factors. Clin. Rheumatol..

[8] Appenzeller S., Bertolo M.B., Costallat L.T. (2004). Cognitive impairment in rheumatoid arthritis. Methods Find. Exp. Clin. Pharmacol..

[9] Meade T., Manolios N., Cumming S.R., Conaghan P.G., Katz P. (2018). Cognitive Impairment in Rheumatoid Arthritis: A Systematic Review. Arthritis Care Res..

[10] Serrano-Castro P.J., Garzón-Maldonado F.J., Casado-Naranjo I., Ollero-Ortiz A., Mínguez-Castellanos A., Iglesias-Espinosa M., Baena-Palomino P., Sánchez-Sanchez V., Sánchez-Pérez R.M., Rubi-Callejon J. (2022). The cognitive and psychiatric subacute impairment in severe COVID-19. Sci. Rep..

[11] Coluccia D., Wolf O.T., Kollias S., Roozendaal B., Forster A., de Quervain D.J. (2008). Glucocorticoid therapy-induced memory deficits: Acute versus chronic effects. J. Neurosci..

[12] Jan J.E., Hill R.H., Low M.D. (1972). Cerebral complications in juvenile rheumatoid arthritis. Can. Med. Assoc. J..

[13] Mena-Vázquez N., Cabezudo-García P., Ortiz-Márquez F., Díaz-Cordovés Rego G., Muñoz-Becerra L., Manrique-Arija S., Fernández-Nebro A. (2021). Evaluation of cognitive function in adult patients with juvenile idiopathic arthritis. Int. J. Rheum. Dis..

[14] Axelrod B.N. (2001). Administration Duration for the Wechsler Adult Intelligence Scale-III and Wechsler Memory Scale-III. Arch. Clin. Neuropsychol. Off. J. Natl. Acad. Neuropsychol..

[15] Shin S.Y., Katz P., Wallhagen M., Julian L. (2012). Cognitive Impairment in Persons with Rheumatoid Arthritis. Arthritis Care Res..

[16] Abeare C.A., Cohen J.L., Axelrod B.N., Leisen J.C., Mosley-Williams A., Lumley M.A. (2010). Pain, Executive Functioning, and Affect in Patients with Rheumatoid Arthritis. Clin. J. Pain.

[17] Faria C.A., Alves H.V.D., Charchat-Fichman H. (2015). The most frequently used tests for assessing executive functions in aging. Dement. Neuropsychol..

[18] Melo L.F.D., Da-Silva S.L. (2012). Neuropsychological Assessment of Cognitive Disorders in Patients with Fibromyalgia, Rheumatoid Arthritis, and Systemic Lupus Erythematosus. Rev. Bras. Reumatol..

[19] Feldmann R., Weglage J., Roth J., Foell D., Frosch M. (2005). Systemic juvenile rheumatoid arthritis: Cognitive function and social adjustment. Ann. Neurol..

[20] Smarr K.L., Keefer A.L. (2011). Measures of Depression and Depressive Symptoms: Beck Depression Inventory-II (BDI-II), Center for Epidemiologic Studies Depression Scale (CES-D), Geriatric Depression Scale (GDS), Hospital Anxiety and Depression Scale (HADS), and Patient Health Questionnaire-9 (PHQ-9). Arthritis Care Res..

[21] Wang Y.P., Gorenstein C. (2013). Psychometric Properties of the Beck Depression Inventory-II: A Comprehensive Review. Rev. Bras. Psiquiatr..

[22] Oxford Grice K., Vogel K.A., Le V., Mitchell A., Muniz S., Vollmer M.A. (2003). Adult norms for a commercially available Nine Hole Peg Test for finger dexterity. Am. J. Occup. Ther..

[23] Feys P., Lamers I., Francis G., Benedict R., Phillips G., LaRocca N., Hudson L.D., Rudick R. (2017). The Nine-Hole Peg Test as a manual dexterity performance measure for multiple sclerosis. Mult. Scler..

[24] Bright P., Hale E., Gooch V.J., Myhill T., van der Linde I. (2018). The National Adult Reading Test: Restandardisation against the Wechsler Adult Intelligence Scale-Fourth edition. Neuropsychol. Rehabil..

[25] Sarkar S., Alam M.M., Das G., Datta S. (2017). Inflammatory Markers and Disease Activity in Juvenile Idiopathic Arthritis. Indian J. Pediatr..

[26] Iwamoto N., Kawakami A., Fujikawa K., Aramaki T., Kawashiri S.Y., Tamai M., Arima K., Ichinose K., Kamachi M., Yamasaki S. (2009). Prediction of DAS28-ESR remission at 6 months by baseline variables in patients with rheumatoid arthritis treated with etanercept in Japanese population. Mod. Rheumatol..

[27] Esteve-Vives J., Batlle-Gualda E., Reig A. (1993). Spanish version of the Health Assessment Questionnaire: Reliability, validity and transcultural equivalency. Grupo para la Adaptación del HAQ a la Población Española. J. Rheumatol..

[28] Mancia G., De Backer G., Dominiczak A., Cifkova R., Fagard R., Germano G., Heagerty A.M., Kjeldsen S.E., Laurent S., Narkiewicz K. (2007). ESH/ESC 2007 Guidelines for the management of arterial hypertension. Rev. Esp. Cardiol..

[29] American Diabetes Association (2010). Diagnosis and classification of diabetes mellitus. Diabetes Care.

[30] Charlson M.E., Pompei P., Ales K.L., MacKenzie C.R. (1987). A new method of classifying prognostic comorbidity in longitudinal studies: Development and validation. J. Chronic Dis..

[31] Hamed S.A., Selim Z.I., Elattar A.M., Elserogy Y.M., Ahmed E.A., Mohamed H.O. (2012). Assessment of biocorrelates for brain involvement in female patients with rheumatoid arthritis. Clin. Rheumatol..

[32] Wallin K., Solomon A., Kåreholt I., Tuomilehto J., Soininen H., Kivipelto M. (2012). Midlife rheumatoid arthritis increases the risk of cognitive impairment two decades later: A population-based study. J. Alzheimers Dis..

[33] Oláh C., Schwartz N., Denton C., Kardos Z., Putterman C., Szekanecz Z. (2020). Cognitive dysfunction in autoimmune rheumatic diseases. Arthritis Res. Ther..

[34] Sood A., Raji M.A. (2021). Cognitive impairment in elderly patients with rheumatic disease and the effect of disease-modifying anti-rheumatic drugs. Clin. Rheumatol..

[35] Lee J.H., Kim G.T., Kim Y.K., Lee S.G. (2018). Cognitive function of patients with rheumatoid arthritis is associated with disease activity but not carotid atherosclerotic changes. Clin. Exp. Rheumatol..

[36] Aimee O., Hersh S.P. (2015). Immunogenetics of Juvenile Idiopathic Arthritis: A Comprehensive Review. J. Autoimmun..

[37] Diaz-Cordovés Rego G., Núñez-Cuadros E., Mena-Vázquez N., Aguado Henche S., Galindo-Zavala R., Manrique-Arija S., Martín-Pedraz L., Redondo-Rodríguez R., Godoy-Navarrete F.J., Fernández-Nebro A. (2021). Adiposity Is Related to Inflammatory Disease Activity in Juvenile Idiopathic Arthritis. J. Clin. Med..

[38] Fuggle N.R., Howe F.A., Allen R.L., Sofat N. (2014). New insights into the impact of neuro-inflammation in rheumatoid arthritis. Front. Neurosci..

[39] Rech J., Hess A., Finzel S., Kreitz S., Sergeeva M., Englbrecht M., Doerfler A., Saake M., Schett G. (2013). Association of brain functional magnetic resonance activity with response to tumor necrosis factor inhibition in rheumatoid arthritis. Arthritis Rheum..

[40] Basile M.S., Ciurleo R., Bramanti A., Petralia M.C., Fagone P., Nicoletti F., Cavalli E. (2021). Cognitive Decline in Rheumatoid Arthritis: Insight into the Molecular Pathogenetic Mechanisms. Int. J. Mol. Sci..

[41] Olah C., Kardos Z., Andrejkovics M., Szarka E., Hodosi K., Domjan A., Sepsi M., Sas A., Kostyál L., Fazekas K. (2019). Assessment of cognitive function in female rheumatoid arthritis patients: Associations with cerebrovascular pathology, depression and anxiety. Rheumatol. Int..

[42] Nerurkar L., Siebert S., McInnes I.B., Cavanagh J. (2019). Rheumatoid arthritis and depression: An inflammatory perspective. Lancet Psychiatry.

[43] Cano-García L., Mena-Vázquez N., Manrique Arija S., Hernández-Sánchez M.D., Segura-Ruiz R., Domínguez-Quesada C., Fernández-Nebro A. (2021). Psychological factors associated with sleep disorders in patients with axial spondyloarthritis or psoriatic arthritis: A multicenter cross-sectional observational study. J. Clin. Nurs..

[44] Cano-García L., Mena-Vázquez N., Manrique-Arija S., Redondo-Rodriguez R., Romero-Barco C.M., Fernández-Nebro A. (2021). Ability to Participate in Social Activities of Rheumatoid Arthritis Patients Compared with Other Rheumatic Diseases: A Cross-Sectional Observational Study. Diagnostics.

[45] Tarakçı E., Arman N., Barut K., Şahin S., Adroviç A., Kasapçopur Ö. (2019). Fatigue and sleep in children and adolescents with juvenile idiopathic arthritis:a cross-sectional study. Turk. J. Med. Sci..

[46] Fair D.C., Rodriguez M., Knight A.M., Rubinstein T.B. (2019). Depression And Anxiety In Patients With Juvenile Idiopathic Arthritis: Current Insights And Impact On Quality Of Life, A Systematic Review. Open Access Rheumatol..

[47] Tarakci E., Yeldan I., Kaya Mutlu E., Baydogan S.N., Kasapcopur O. (2011). The relationship between physical activity level, anxiety, depression, and functional ability in children and adolescents with juvenile idiopathic arthritis. Clin. Rheumatol..

[48] Sweeney M., Carpenter L., de Souza S., Chaplin H., Tung H., Caton E., Galloway J., Cope A., Yates M., Nikiphorou E. (2022). The impact of COVID-19 on clinical care, self-management and mental health of patients with inflammatory arthritis. Rheumatol. Adv. Pract..

