# Supplementary material for: Longitudinal Study of Cognitive Functioning in Adults with Juvenile Idiopathic Arthritis
Source: Biomedicines. 2022 Jul 18;10(7):1729. doi: 10.3390/biomedicines10071729 (PMC9312867; doi:10.3390/biomedicines10071729)
Supplement: Supplementary file 1 [file biomedicines-10-01729-s001.zip › biomedicines-1786399-supplementary.pdf]

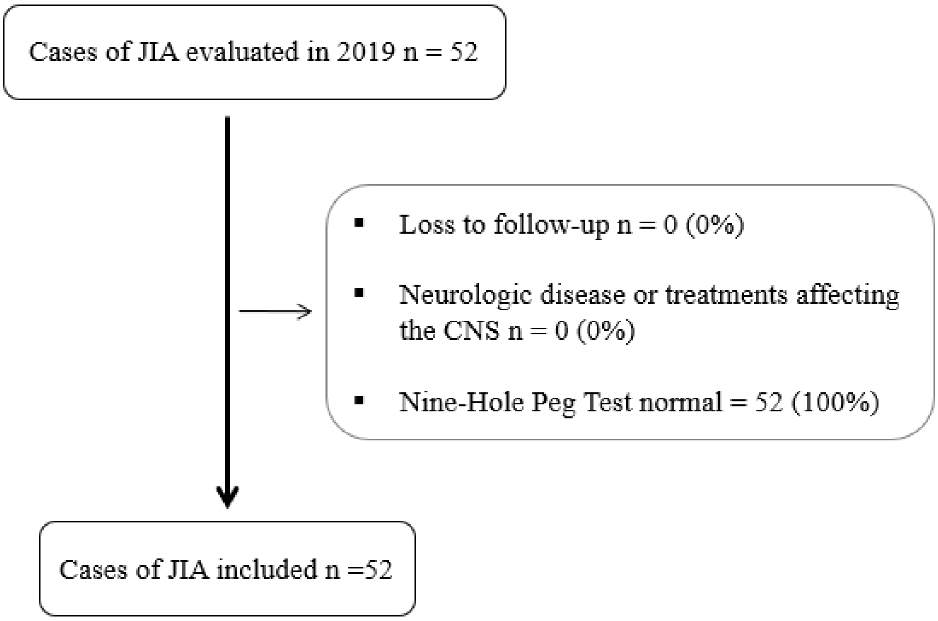

Figure S1. Patient flowchart.

A)

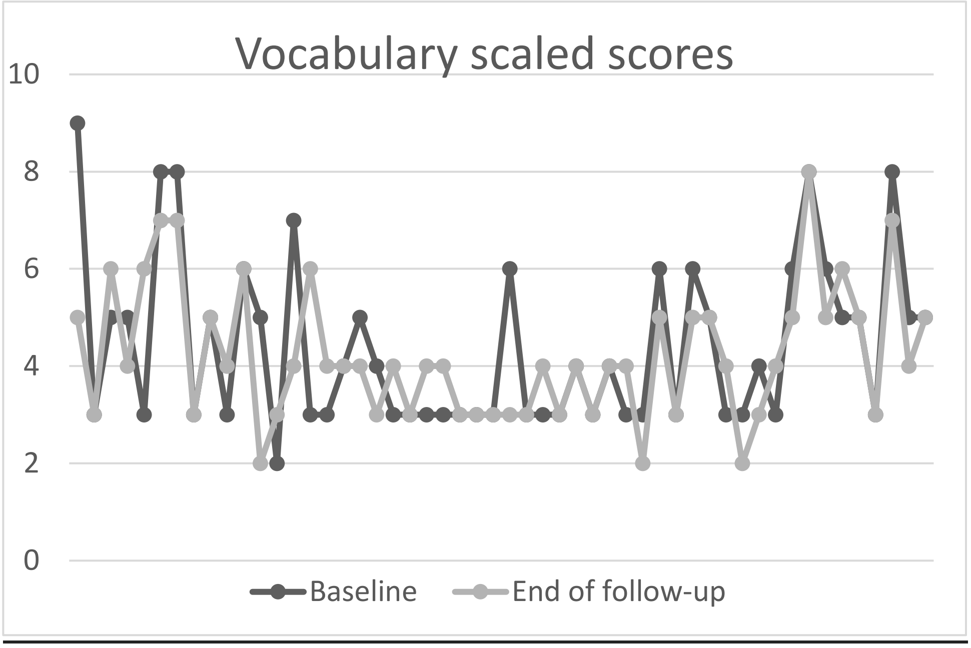

B)

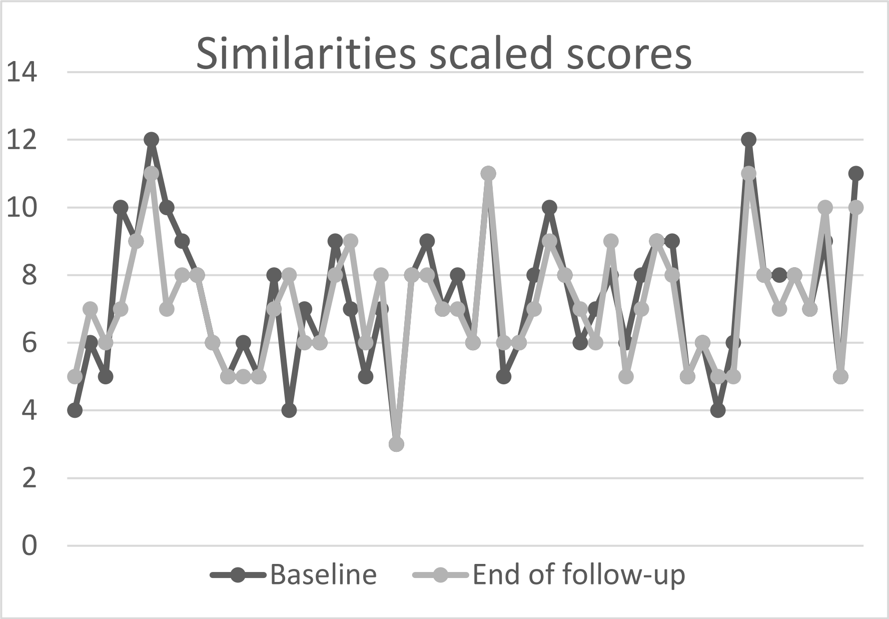
Similarities scaled scores
14
12
10
8
6
4
2
0
Baseline
End of follow-up

C)

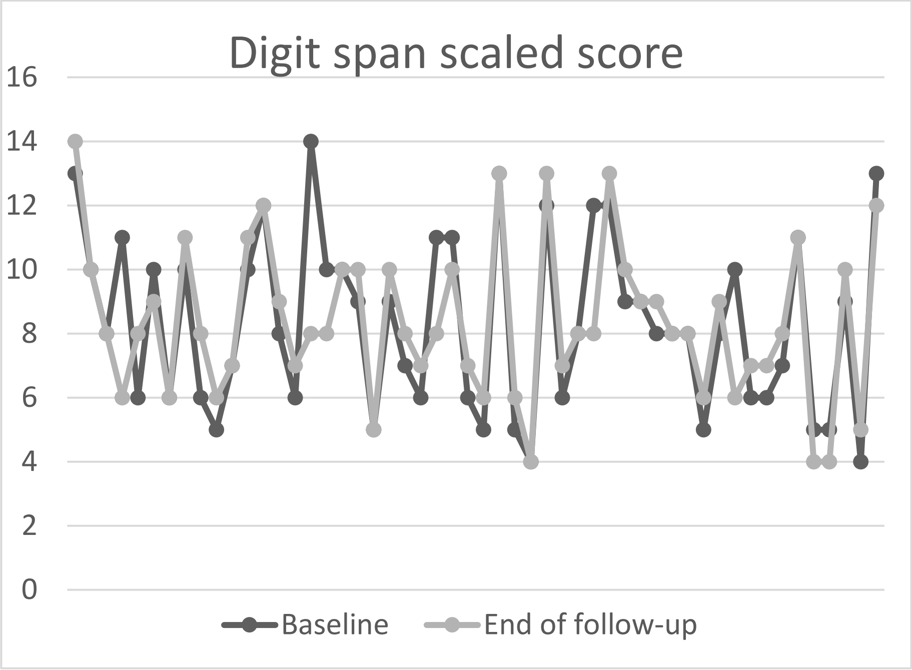
Digit span scaled score
16
14
12
10
8
6
4
2
0
Baseline
End of follow-up

D)

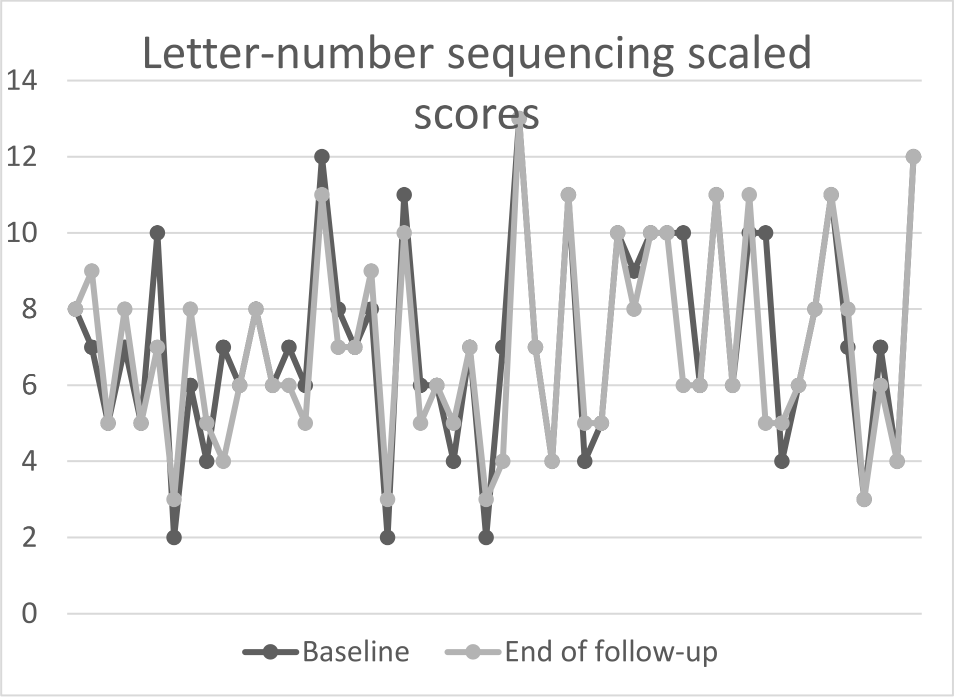

E)

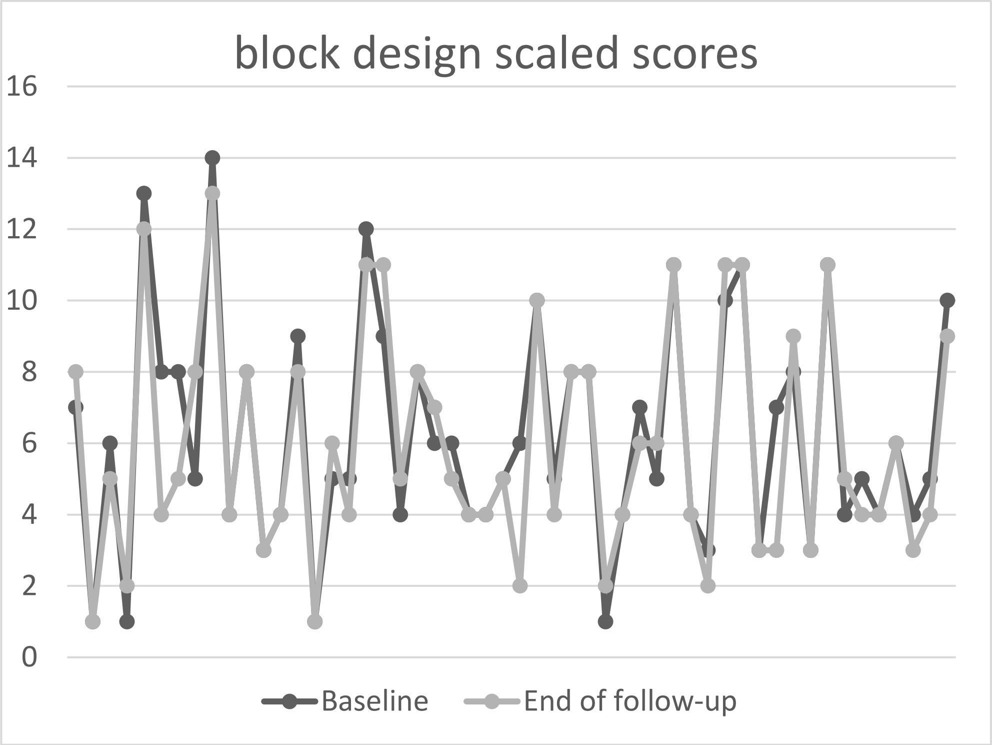

Figure S2. Scaled Scores.(**A**): Vocabulary scaled scores for each patient; (**B**): Similarities scaled scores for each patient; (**C**): Digit span scaled score for each patient; (**D**): Letter-number sequencing scaled scores for each patient; (**E**): block design scaled scores for each patient.

Table S1. Characteristics of patients with JIA and cognitive decline.

Patient	Age at V24 (years)	Treatment at V24	Average CRP (mg/dl)	Depression by BDI (number)	Tests affected
Patient 1	29.7	Sulfasalazine	14.3	8.0	Vocabulary
Patient 2	28.4	No DMARDs	3.23	15.0	Letter-number sequencing, block design
Patient 3	22.0	Etanercept	1.82	8.0	Digit span
Patient 4	19.6	Methotrexate	3.20	12.0	Similarities, digit span
Patient 5	24.0	No DMARDs	14.1	13.0	Digit span
Patient 6	21.5	Methotrexate	6.28	5.0	Digit span
Patient 7	40.1	Adalimumab	12.9	15.0	Similarities, block design
Patient 8	25.8	Etanercept	4.0	12.0	Digit span, letter-number sequencing
Patient 9	18.6	Leflunomide + golimumab	15.0	14.0	Vocabulary, block design, letter-number sequencing
Patient 10	17.4	Etanercept	3.7	9.0	Block design
Patient 11	18.2	No DMARDs	22.2	4.0	Digits
Patient 12	20.2	No DMARDs	9.6	4.0	Vocabulary
Patient 13	17.9	Methotrexate	2.9	0	Letter-number sequencing
Patient 14	21.9	No DMARDs	3.45	2.0	Vocabulary
Patient 15	27.5	No DMARDs	2.28	6.0	Letter-number sequencing

Abbreviations; CRP: C-reactive protein; DMARD: disease-modifying antirheumatic drug.

Table S2. Univariate and multivariate logistic regression of the characteristics of JIA associated with cognitive decline excluding 7 patients with treatment change.

Variable	Univariate HR (95% CI)	Multivariate HR (95% CI)	p value
Age, years	0.808 (0.921-1.112)		
Female sex	0.960 (0.268-3.436)		

Educational level*	0.157 (0.025-0.977)		
Body mass index (kg/m^2)	1.152 (1.004-1.323)		
Average C-reactive protein (mg/dl)	1.392 (1.089-1.779)	1.261 (1.006-1.625)	0.041
Treatment with biologics	0.212 (0.059-0.764)	0.180 (0.033-0.971)	0.045
Depression by BDI ≥ 14	3.338 (1.357-8.213)	2.852 (1.079-1.536)	0.035

*Educational level: higher or university vs. basic. Nagelkerke R2 = 0.481. The variables included in the equation were age, sex, educational level, body mass index, C-reactive protein, treatment with biologics, depression by BDI ≥ 14.
